# Socioeconomic Position, Type 2 Diabetes and Long-Term Risk of Death

**DOI:** 10.1371/journal.pone.0124829

**Published:** 2015-05-05

**Authors:** Else-Marie Dalsgaard, Mette V. Skriver, Annelli Sandbaek, Mogens Vestergaard

**Affiliations:** 1 Department of Public Health, Section for General Practice, Aarhus University, Denmark; 2 Department of Public Health, Section for Health Promotion and Health Services, Aarhus University, Denmark; 3 Department of Public Health, Section for General Practice and Research Unit for General Practice, Aarhus University, Denmark; Public Health Agency of Canada, CANADA

## Abstract

**Background:**

Both socioeconomic position (SEP) and type 2 diabetes have previously been found to be associated with mortality; however, little is known about the association between SEP, type 2 diabetes and long-term mortality when comorbidity is taken into account.

**Methods:**

We conducted a population-based cohort study of all Danish citizens aged 40-69 years with no history of diabetes during 2001-2006 (N=2,330,206). The cohort was identified using nationwide registers, and it was followed for up to 11 years (mean follow-up was 9.5 years (SD: 2.6)). We estimated the age-standardised mortality rate (MR) and performed Poisson regression to estimate the mortality-rate-ratio (MRR) by educational level, income and cohabiting status among people with and without type 2 diabetes.

**Results:**

We followed 2,330,206 people for 22,971,026 person-years at risk and identified 139,681 individuals with type 2 diabetes. In total, 195,661 people died during the study period; 19,959 of these had type 2 diabetes. The age-standardised MR increased with decreasing SEP both for people with and without diabetes. Type 2 diabetes and SEP both had a strong impact on the overall mortality; the combined effect of type 2 diabetes and SEP on mortality was additive rather than multiplicative. Compared to women without diabetes and in the highest income quintile, the MRR’s were 2.8 (95%CI 2.6, 3.0) higher for women with type 2 diabetes in the lowest income quintile, while diabetes alone increased the risk of mortality 2.0 (95%CI 1.9, 2.2) times and being in the lowest income quintile without diabetes 1.8 (95%CI 1.7,1.9) times after adjusting for comorbidity. For men, the MRR’s were 2.7 (95%CI 2.5,2.9), 1.9 (95%CI 1.8,2.0) and 1.8 (95%CI 1.8,1.9), respectively.

**Conclusion:**

Both Type 2 diabetes and SEP were associated with the overall mortality. The relation between type 2 diabetes, SEP, and all-cause mortality was only partly explained by comorbidity.

## Introduction

Type 2 diabetes is a major public health problem; its incidence is rising and the mortality from diabetes is high [1]. The prevalence of type 2 diabetes is higher among people with a lower socioeconomic position (SEP) than among people with a higher SEP [2, 3], and the burden of complications increases with declining SEP [4]. It has been suggested that both personal factors and structural factors may explain why the burden of type 2 diabetes is higher among people with a lower SEP [5].

A few studies have compared the association between SEP and mortality in people with type 2 diabetes and people without type 2 diabetes [6–9]. These studies found that the all-cause mortality increases with decreasing SEP in both groups, but the strongest association was seen in people without diabetes. The excess absolute mortality among people with lower SEP and type 2 diabetes constitutes a major public health burden [8,9]. In Denmark, the relative income disparity is low, diabetes care is well-organized, and access to the health care system is free of charge. In this Danish context, we conducted a nationwide cohort study in which we aimed at examining the association between SEP, type 2 diabetes and the long-term mortality while taking comorbidity into account.

## Materials and Methods

We conducted a population-based cohort study based on nationwide Danish registers. All people with no history of diabetes aged 40–69 years between 1 January 2001 and 31 December 2006 were identified from the Danish Civil Registration System [10].

### Type 2 diabetes

Incident cases of diabetes throughout the period 1 January 2001 to 31 December 2009 were identified from the Danish National Diabetes Register (NDR) [11]. Cases were identified as having diabetes if they fulfilled at least one of the following criteria: 1) registered in the National Patient Register [12] with a diagnosis of diabetes defined according to the diagnostic codes of the 10^th^ edition of the International Classification of Diseases (ICD-10): DE10-14, DH36.0, DO24 (excluding DO24.4), or ICD8: 249 or 250; 2) registered in the National Health Service Registry [14] with chiropody (patients with diabetes), five blood glucose measurement within one year, or two blood glucose measurements per year in five consecutive years; or 3) registered in the Danish National Prescription Registry [14] with two redemptions of glucose-lowering drugs recorded within six months or a second purchase of prescribed insulin. The NDR [11] does not distinguish between type 1 and type 2 diabetes. As we included only people registered after the aged of 40 years in the NDR, we regard our population to have type 2 diabetes.

### Socioeconomic position

SEP was measured in terms of educational level, income level, and cohabitation status. The data were collected from the Danish Integrated Database for Labour Market Research [15] for the year before the case entered the study. Educational level was categorised according to UNESCO’s International Standard Classification of Education [16], and educational levels were divided into three groups according to the years of schooling: 1) low educational level: ≤ 10 years, 2) middle educational level: > 10 and ≤ 15 years, and 3) high educational level: > 15 years. Income level was categorised according to the OECD-modified equivalence-weighted income scale [17]; in this scale, the first adult in the household is given the weight 1, the second adult the weight 0.5, and each child the weight 0.3. Income level was divided into three groups: the lowest 20% of the income earners, the medium 60% of the income earners, and the highest 20% of the income earners. Cohabitation status was derived from the Danish Civil Registration System [10] and categorised into two groups: cohabiting people and people living alone.

### Mortality

We collected information on mortality before 31 December 2011 from the Danish Civil Registration System [10].

### Covariates

We collected information on age and sex from the Danish Civil Registration System [10] and hospital discharge diagnoses from the Danish National Patient Register [13]. We identified people with a history of cardiovascular disease (CVD) or cancer if they had been given an ICD-10 diagnosis for CVD (ICD-10: I00-I79 and I90-I94) or for cancer (ICD-10: C00-C99) during the last five years before inclusion. Furthermore, during follow-up we registered any instance of CVD, cancer, and other diseases (defined as number of diseases other than CVD and cancer from the list of diseases included in the Charlson Comorbidity Index [18]).

### Statistical analyses

The study population was characterised using the chi-square test and the t-test. The age-standardised mortality rate (MR) for each sex by SEP was calculated for people with and without type 2 diabetes using the total study population divided into five-year age groups as our standard population. Diabetes was treated as a time-dependent variable. Thus, all people were included in the non-diabetes cohort when they were enrolled into the study, and subsequently transferred to the diabetes cohort on the day when they were registered with diabetes in the NDR. The study population was followed until the date of migration, the date of death, or 31 December 2011, whichever came first. Poisson regression analysis was performed to estimate the mortality rate ratio (MRR); the reference group consisted of those people without diabetes who had the highest educational level, the highest income level, or were cohabiting, respectively. Separate analyses were performed for each sex. Model 1 was adjusted for age (in five-year age groups), duration of diabetes (0–2, 2–4, 4–6, >6 years), and calendar year; and an interaction term between the SEP variable and the birth year (in five-year groups) was included. Model 2 was further adjusted for CVD and cancer during the last five years before inclusion. Model 3 was further adjusted for time-dependent comorbidity (CVD, cancer, and other diseases according to the Charlson Comorbidity Index) during follow-up. In analyses with educational and income levels as exposures, we added cohabitation status to the model, while educational level was added to the model where cohabitation status was considered as exposure. Analyses were repeated with exclusion of the first year after inclusion.

We tested for interaction between each SEP variable and diabetic status to measure the extent to which the effect of the two factors together exceeded the effect of each individually factor. As interaction depends on the mode chosen, we tested both on a multiplicative scale and on an additive scale. To evaluate whether there was interaction on the multiplicative scale, a model with an interaction term between the two variables was applied. To evaluate interaction on the additive scale we estimated the additive interaction by using the risk ratios instead of the risks [19]. We calculated the attributable proportion due to interaction (AP). The AP is a measure of the excess MRR (for people with both type 2 diabetes and low SEP), which was not explained by the independent effects of type 2 diabetes or low SEP. The AP was calculated by subtracting the difference in MRRs between people with and without diabetes from the difference in MRRs between people with low SEP and diabetes and people with low SEP without diabetes and dividing this by the MRR for people with both diabetes and low SEP (e.g. AP_interaction_ = [(MRR_lowSEP+DM—_MRR_lowSEP_)–(MRR_DM_—MRR_background_) /MRR_DM+lowSEP_] [20]. All analyses were performed using Stata 12.1.

Data were handled according to rules from the Danish Data Protection Agency (Jr.nr: 2014–54–0704). In Denmark, ethical approval is not required for de-identified register-based research.

## Results

A total of 2,330,206 people without a history of diabetes were enrolled into the study; 139,681 of these developed type 2 diabetes during the study period (Table 1). The population was followed for up to 11 years; the mean follow-up was 9.5 years (standard deviation (SD): 2.6) for the study population and 5.7 years (SD: 2.7) for the diabetes population. People with type 2 diabetes were more likely than the total study population to have a lower educational level, a lower income level, and to be living alone. A history of comorbid disease at the time of inclusion or development of comorbid disease during follow-up was more common among people with type 2 diabetes than among the total study population.

**Table 1:** Characteristics of people with and without diabetes and the general population aged 40–69 years, 2001–06.

	Diabetes	%	Population	%
N	139,681	6	2,330,206	
Sex				
Men	78,209	56	1,160,649	49
Women	61,472	44	1,169,557	50
Age, mean (SD)	55.2 (8.5)		50.7 (9.1)	
Educational level				
> 15 years	16,541	11	459,710	19
10–15 years	61,081	43	1,105,783	47
≤ 10 years	62,059	44	764,721	32
Income level				
80 percentile	21,419	15	466,037	20
20–80 percentile	84,412	60	1,398,126	60
20 percentile	33,850	24	466,042	20
Cohabitation status				
Cohabitant	100,384	71	1,759,990	75
Living alone	39,297	28	570,216	24
Death before follow-up	19,947	14	195,198	8
Age at death, mean (SD)	67.0 (7.9)		64.6 (8.8)	
CVD before study start	27,342	19	123,636	5
Cancer before study start	7,035	4	55,809	2
CVD during follow-up	40,476	28	382,715	16
Cancer during follow-up	17,269	12	220,498	9
Other diseases during follow-up	22,172	15	270,844	11

**Fig 1:**
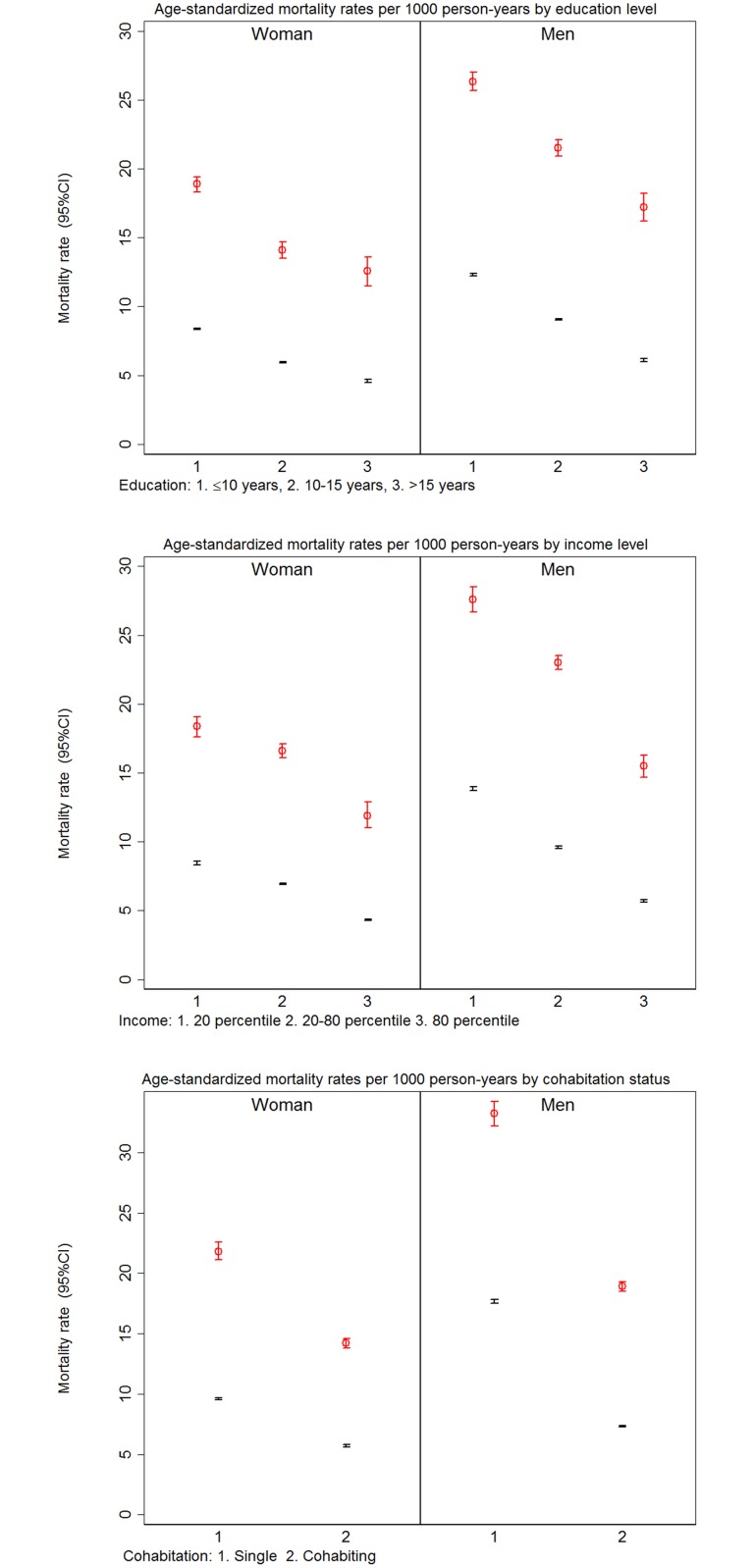
Age-standardized mortality rates according to educational level, income level and cohabitation status among people with and without diabetes in the Danish population aged 40–69 years at inclusion, mean follow-up 9.5 years. Red: People with diabetes, Blue: People without diabetes.

A total of 195,661 people died during the study period; 19,959 (14% of people who had a history of type 2 diabetes). There was a strong association between SEP, type 2 diabetes and the overall mortality. For both people with and without type 2 diabetes, the age-standardised MRs increased with decreasing educational level and decreasing income level, and the MRs were higher for people living alone than for cohabiting people (Fig 1). The excess mortality for people with type 2 diabetes compared with people without diabetes was stable across the SEP indicators; for example, 14 per 1000 person-years (95% CI 12 to 16) for men and 11 per 1000 person-years (95% CI 9 to 12) for women with the lowest educational level, 13 per 1000 person-years (95% CI 11 to 14) for men and 8 per 1000 person-years (95% CI 7 to 9) for women with a medium educational level, and 11 per 1000 person-years (95% CI 10 to 12) for men and 8 per 1000 person-years (95% CI 7 to 9) for women with the highest educational level (Fig 1).

People with type 2 diabetes and the lowest SEP (lowest educational level, lowest income level, or living alone) had a more than two-fold higher MRR than people without diabetes and the highest SEP (highest educational level, highest income level, or cohabiting) (Table 2). In general, associations between the SEP variables and mortality were similar for both sexes, but men living alone tended to have higher MRRs than women living alone. We found that the AP for interaction between type 2 diabetes and each of the three SEP indicators (education, income, and cohabitation) were not statistical different from zero for women (S1 Table) indicating that the combined effects of SEP and type 2 diabetes on mortality among women were additive (ie, _MRRlowSEP+DM = MRRlowSEP + MRRDM—MRRref:_ or 2.7 = 1.7+2.1–1 from Table 2 for women and education). Among men the AP’s were close to zero. However, for educational level and cohabitating status they were statistical significant and negative (S1 Table).

**Table 2:** MRR for all-cause mortality among people with and without diabetes in the Danish population aged 40–69 years at inclusion, mean follow-up: 9.5 years.

	Model 1		Model 2		Model 3	
	Without diabetes	Diabetes	Without diabetes	Diabetes	Without diabetes	Diabetes
**Women**						
**Educational level**						
> 15 years	Ref.	2.2 (2.0,2.4)	Ref.	2.1 (1.9,2.3)	Ref.	2.1 (1.9,2.3)
10–15 years	1.3 (1.3,1.4)	2.5 (2.3,2.6)	1.3 (1.3,1.4)	2.3 (2.1,2.5)	1.3 (1.2,1.3)	2.2 (2.0,2.3)
≤ 10 years	1.7 (1.6,1.7)	2.8 (2.7,3,0)	1.7 (1.6,1.8)	2.7 (2.6,2.9)	1.7 (1.6,1.8)	2.7 (2.5,2.8)
**Income level**						
80 percentile	Ref.	2.3 (2.1,2.5)	Ref.	2.2 (2.0,2.3)	Ref.	2.0 (1.9,2.2)
20–80 percentile	1.4 (1.4,1.5)	2.7 (2.5,2.9)	1.5 (1.4,1.6)	2.5 (2.4,2,7)	1.5 (1.4,1.5)	2.5 (2.3,2.7)
20 percentile	1.7 (1.6,1.8)	2,8 (2.6,3.0)	1.7 (1.7,1.8)	2.7 (2.5,2.9)	1.8 (1.7,1.9)	2.8 (2.6,3.0)
**Cohabitation status**						
Cohabitant	Ref.	1.9 (1.8,2.0)	Ref.	1.8 (1.7,1.9)	Ref.	1.8 (1.7,1.8)
Living alone	1.5 (1.5,1.5)	2.5 (2.3,2.6)	1.5 (1.4,1.5)	2.3 (2.2,2.4)	1.4 (1.4,1.5)	2.2 (2.1,2.4)
**Men**						
**Educational level**						
> 15 years	Ref.	2.2 (2.0,2.3)	Ref.	2.0 (1.9,2.2)	Ref.	1.9 (1.8,2,1)
10–15 years	1.3 (1.3,1.4)	2.5 (2.3,2.6)	1.3 (1.3,1.4)	2.3 (2.1,2.4)	1.3 (1.3,1.4)	2.15 (2.0,2.3)
≤ 10 years	1.5 (1.4,1.5)	2.4 (2.3,2.6)	1.5 (1.4,1.5)	2.3 (2.2,2.4)	1.5 (1.5,1.6)	2.3 (2.2,2.4)
**Income level**						
80 percentile	Ref.	2.2 (2.1,2.3)	Ref.	2.0 (1.9,2.2)	Ref.	1.9 (1.8,2.0)
20–80 percentile	1.5 (1.5,1.6)	2.8 (2.6,3.0)	1.6 (1.5,1.6)	2.6 (2.4,2.7)	1.5 (1.5,1.6)	2.4 (2.3,2.6)
20 percentile	1.8 (1.8,1.9)	2.9 (2.8,3.1)	1.8 (1.8,1.9)	2.7 (2.6,2.9)	1.8 (1.8,1.9)	2.7 (2.5,2.9)
**Cohabitation status**						
Cohabitant	Ref.	2.0 (2.0,2.1)	Ref.	1.8 (1.8,1.9)	Ref.	1.8 (1.7,1.9)
Living alone	1.8 (1.8,1.9)	2.7 (2.6,2.9)	1.9 (1.8,1.9)	2.6 (2.5,2.7)	1.9 (1.9,2.0)	2.6 (2.4,2.7)

Model 1. Adjusted for age, duration of diabetes, calendar time. Model 2. Further adjusted for CVD and cancer before study start. Model 3. Further adjusted for CVD and cancer before study start and CVD, cancer and no. of other diseases according to Charlson Comorbidity Index during follow-up. Reference: Highest SEP-level among people without diabetes, Interaction between SEP and diabetic status, p<0.001

The MRRs for SEP and type 2 diabetes were attenuated by adjustments for a history of CVD and cancer at the time of inclusion (Model 2) as well as by further adjustments for CVD, cancer, and other diseases that developed during follow-up (Model 3). The MRRs did, however, remain high and statistically significant. In sub-analyses, we eliminated the first year after inclusion for people with type 2 diabetes, but this did not change the estimates (data not shown).

Information on the SEP variables were missing in less than 3% of the study population, which was due mostly to missing information on education. People with missing information on education were more likely to be younger, to be men, to be immigrants, to have lower or middle income, and to live alone. We performed sensitivity analyses in which people with missing information on education were classified into the lowest educational category and the highest educational category. The association between SEP variables, diabetes and mortality were moderately attenuated when the missing data were included into the highest level, whereas no change was seen in the estimates when the missing data were included into the lowest level.

## Discussion

In this nationwide cohort study, we found that type 2 diabetes and socioeconomic position had a strong impact on the overall mortality. The MRs increased consistently with decreasing levels of education and income, and among people living alone compared with cohabiting people; this increase was seen both among people with and without diabetes. The excess mortality of people with type 2 diabetes relative to those without diabetes was stable across SEPs. We found that the combined effects of the SEP variables and type 2 diabetes on mortality among women were additive rather than multiplicative while the combined effect among men were both lower than the sum and lower than the product of the two variables for educational level and cohabiting status. The association between the SEP variables and mortality was only slightly attenuated when we adjusted for a history of comorbidity at inclusion and for comorbidity that developed during follow-up, but it remained strong for educational level, income level, and cohabitation status.

We conducted a large population-based cohort study of all people in Denmark without diabetes aged 40 to 69 years between 2001 and 2006, and we followed the cohort for up to 11 years without loss to follow-up. We had complete information on SEP for 97% of the population, and sensitivity analyses did not change the results. Thus, bias due to the selection of study participants or loss to follow-up cannot explain our results. The large size of the cohort provided precise estimates and sufficient power to stratify the multivariate analyses.

The use of data from Danish registers allowed us to obtain information on all the included elements at the level of the individual person. The Danish National Diabetes Register covers the entire Danish population; its sensitivity compared with general practice records is 91%, and it has a positive predictive value of 89% [11]. There is no reason to believe that enrolment in the register differed between the SEP levels, and any misclassification of diabetes is therefore most likely non-differential. Even though the register does not distinguish between type 1 and type 2 diabetes, we regard people with diabetes in this study to have type 2 diabetes as we enrolled only people diagnosed after the age of 40 years.

Socioeconomic data from the Danish Integrated Database for Labour Market Research [15] are regarded as valid, and they were collected prospectively and did not depend on people’s memory. Misclassification is therefore most likely non-differential and bias will be towards the null value.

We included only people with incident diabetes to ensure a homogenous population and to reduce the risk of confounding by severity of diabetes disease. We enrolled incident cases of diabetes on the basis of the date of registration in the NDR [11], even if the disease might have been present several years before the person was diagnosed [21]. We controlled for calendar time and duration of diabetes as both factors may influence mortality among people with diabetes [22]. The overall mortality of the Danish population has generally declined in recent years. This decrease has been particularly prominent among people with type 2 diabetes and has presumably been achieved owing to a strengthened diagnostic effort and focus on early treatment initiation [23].

Residual confounding factors remain an issue even though we adjusted for several confounders. Thus, we had no information on lifestyle factors such as physical activity, body mass index, and smoking status; and we had no information on intermediary measures (e.g. HbA_1c_, cholesterol and blood pressure levels) or subclinical morbidity.

In line with findings from previous studies, we found that the mortality among people with type 2 diabetes increased with decreasing SEP [6–9,24–26]. In the US National Health Interview Survey, Dray-Spira et al. [9] found a 28% higher mortality among people with type 2 diabetes and a low educational level than among those with a higher educational level. Similar results have been found in Scotland [8], Finland [27], and two North Italian cities [6], although no association was found among women with type 2 diabetes in the Italian study.

Only a few studies have compared the association between SEP and mortality in people with type 2 diabetes and in people without diabetes [6–9]. Our findings of higher absolute MRs among people with type 2 diabetes and increasing MR with declining SEP support the findings of others [8,9], and our results underline the magnitude of the mortality burden among people with type 2 diabetes and a low SEP. We found a more than two-fold higher MRR among people with type 2 diabetes and a low SEP than among people without diabetes and the highest SEP. Our reference group was chosen to underscore the impact of having both type 2 diabetes and a low SEP. Other studies present a stronger association among the population without diabetes when comparing the mortality and different SEP indicators [6–9]. These differences are due mainly to the choice of reference group; Walker et al. [8] compared the MRR among people with and without type 2 diabetes from the same area-based deprivation level and found the strongest association in the least deprived group. Dray-Spira et al. [9] chose the highest educational level among people with and without type 2 diabetes as reference group. Even so, both our study and other studies found the combined effect of SEP and type 2 diabetes to be additive i.e. less than multiplicative [6,8,9]. The stronger association between SEP and mortality found among people without diabetes in all these studies was suggested to be explained by better cardiovascular control among deprived people with type 2 diabetes than among deprived people without diabetes owing to the intensive diabetes treatment regimens with regular control of HbA_1c_, cholesterol, and blood pressure and owing to multifactorial drug treatment [8,9]. This is supported by recent studies, including a Danish study, which found that risk factors were controlled to the same extent among people with type 2 diabetes regardless of their SEP [28–30].

We examined three SEP indicators (education, income, and cohabitation status) which illustrate different aspects of SEP [31,32]. The three indicators all showed an inverse relation between SEP and mortality among people with and without type 2 diabetes and this emphasizes the social gradient in mortality. This is in line with the findings of other studies [27,33], which have examined the ability of different indicators of SEP to predict mortality among people with type 2 diabetes.

Our finding of only a moderate decline in the association between the SEP indicators, type 2 diabetes and mortality when adjusting for comorbidity both before inclusion and during follow-up is notable and it is supported by the findings of a recent US study [33]. While type 2 diabetes is often diagnosed when a person is hospitalized for other reasons, it has been found that people with type 2 diabetes had an increased risk of dying in the first year after diagnosis [23]. Furthermore, it has been suggested that people with low SEP are often diagnosed later in the disease trajectory and consequently suggested to have more complications, including higher mortality [9]. However, our findings do not support this suggestion as we found the association to be similar after having excluded from the analyses the first year after the diabetes diagnosis.

Both type 2 diabetes and income, education and cohabitating status had a significant effect on all-cause mortality. Mortality rates were higher among people with low SEP than among those with high SEP both among people with and people without type 2 diabetes. All-cause mortality was only partly explained by comorbidity.

## Supporting Information

S1 Table Attributable proportion due to interaction between type 2 diabetes and the SEP variable (DOCX)
