# Supplementary material for: Socioeconomic Position, Type 2 Diabetes and Long-Term Risk of Death
Source: PLoS One. 2015 May 5;10(5):e0124829. doi: 10.1371/journal.pone.0124829 (PMC4420496; doi:10.1371/journal.pone.0124829)
Supplement: S1 Table — (DOCX) [file pone.0124829.s001.docx]

S1 Tabel Attributable proportion due to interaction between type 2 diabetes and the SEP variable

|  | Model 1 | Model 2 | Model 3 |
| --- | --- | --- | --- |
|  |  |  |  |
| **Women** |  |  |  |
| Education | -0.04 (-0.11,0.04) | -0.03 (-0.10,0.05) | -0.06 (-0.13,0.02) |
| Income | -0.07 (-0.15,0.01) | -0.07 (-0.14,0.00) | -0.04 (-0.11,0.04) |
| Cohabiting | 0.01 (-0.04,0.06) | 0.00 (-0.04,0.05) | 0.01 (-0.03,0.06) |
| **Men** |  |  |  |
| Education | -0.08 (-0.14,-0.02) | -0.10 (-0.16,-0.03) | -0.08 (-0.14,-0.02) |
| Income | -0.04 (-0.09,0.02) | -0.06 (-0.12,0.00) | -0.02 (-0.08,0.03) |
| Cohabiting | -0.04 (-0.08,-0.01) | -0.04 (-0.07,0.00) | -0.04 (-0.08,-0.01) |

Model 1. Adjusted for age, duration of diabetes, calendar time. Model 2. Further adjusted for CVD and cancer before study start. Model 3. Further adjusted for CVD and cancer before study start and CVD, cancer and no. of other diseases according to Charlson Comorbidity Index during follow-up.
